# Supplementary material for: Myeloperoxidase Gene Deletion Causes Drastic Microbiome Shifts in Mice and Does Not Mitigate Dextran Sodium Sulphate-Induced Colitis
Source: Int J Mol Sci. 2024 Apr 11;25(8):4258. doi: 10.3390/ijms25084258 (PMC11050303; doi:10.3390/ijms25084258)
Supplement: Supplementary file 1 [file ijms-25-04258-s001.zip › ijms-2905889-supplementary.pdf]

Myeloperoxidase Gene Deletion Causes Drastic Microbiome Shifts in Mice and Does Not Mitigate Dextran Sodium Sulfate-Induced Colitis

Supplementary Figures and legends

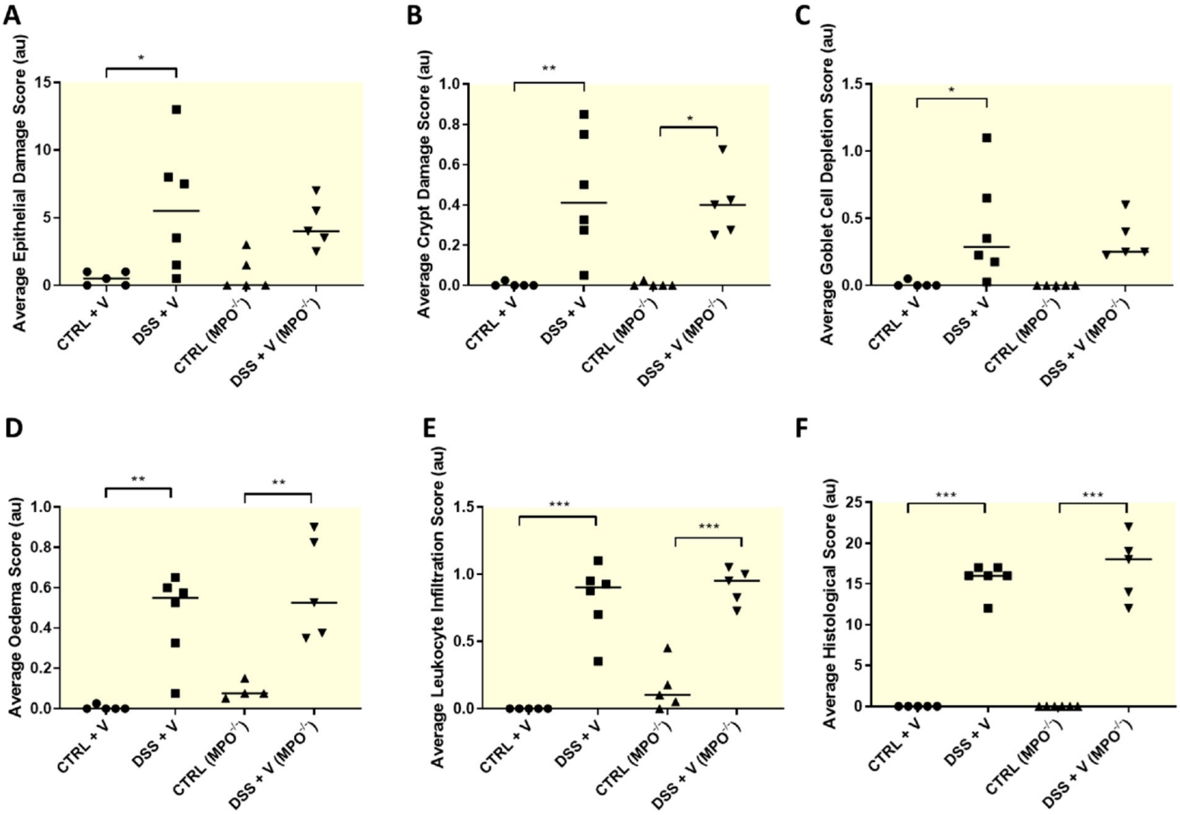

Supplementary Figure S1. Histopathological parameters scored on colons subjected to chronic DSS colitis. Colons from mice subjected to chronic DSS colitis were resected at model endpoint (D=63) and stained with haematoxylin & eosin. Semi-quantitative scoring of 20 random fields of view at 400x microscopic magnification (a score of between 0-2 per field of view for each parameter) of several histopathological parameters including A) epithelial damage, B) crypt damage, C) goblet cell depletion, D) level of oedema and E) level of leukocyte infiltration were assessed as markers of colitis severity. Enumeration of aforementioned scores yielded an F) average histological score for each experimental group. CTRL = control. DSS = dextran sodium sulfate. MPO KO = myeloperoxidase gene deficient. Data represented as mean ± SD. n = 5 Wt CTRL, n = 6 Wt DSS, n = 4 MPO $^{-/-}$ CTRL and n = 5 MPO $^{-/-}$ DSS. * $p \leq 0.05$, ** $p \leq 0.01$, *** $p \leq 0.001$.

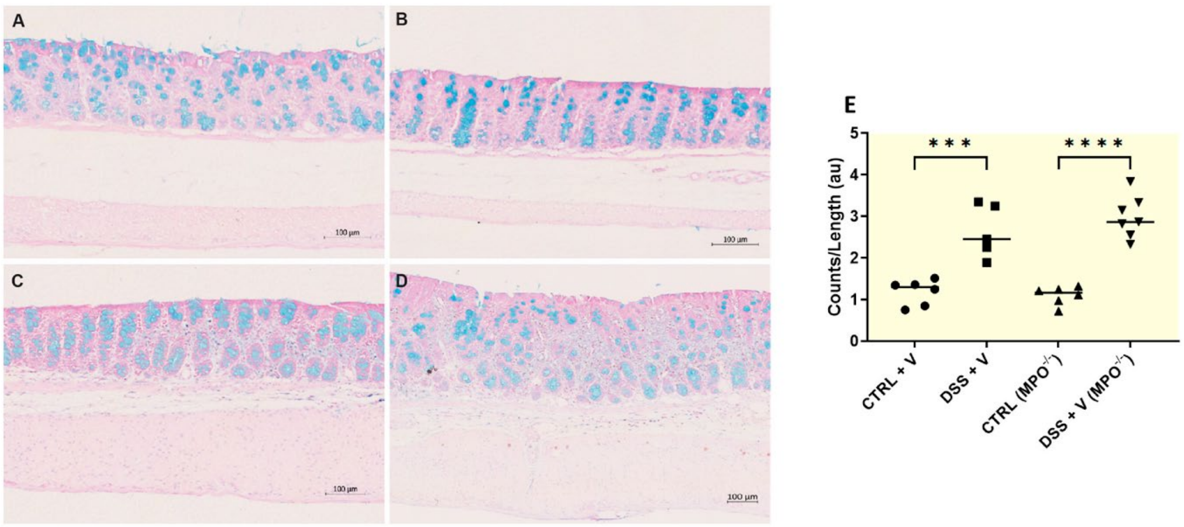

Supplementary Figure S2. Representative alcian blue and Safranin O staining of colonic section stains with quantification. Representative colon sections stained with Alcian blue and Safranin O after 63-day chronic colitis model of groups A) CTRL, B) MPO KO-CTRL, Wt-DSS, C) and D) MPO KO-DSS. E) Quantitative assessment of positive alcian blue and Safranin staining of mucin in colons subjected to chronic DSS colitis. CTRL = control. DSS = dextran sodium sulfate. MPO KO = myeloperoxidase gene deficient. Data represented as mean ± SD. n = 6, all groups. ***$p \leq 0.001$ & ****$p \leq 0.0001$.

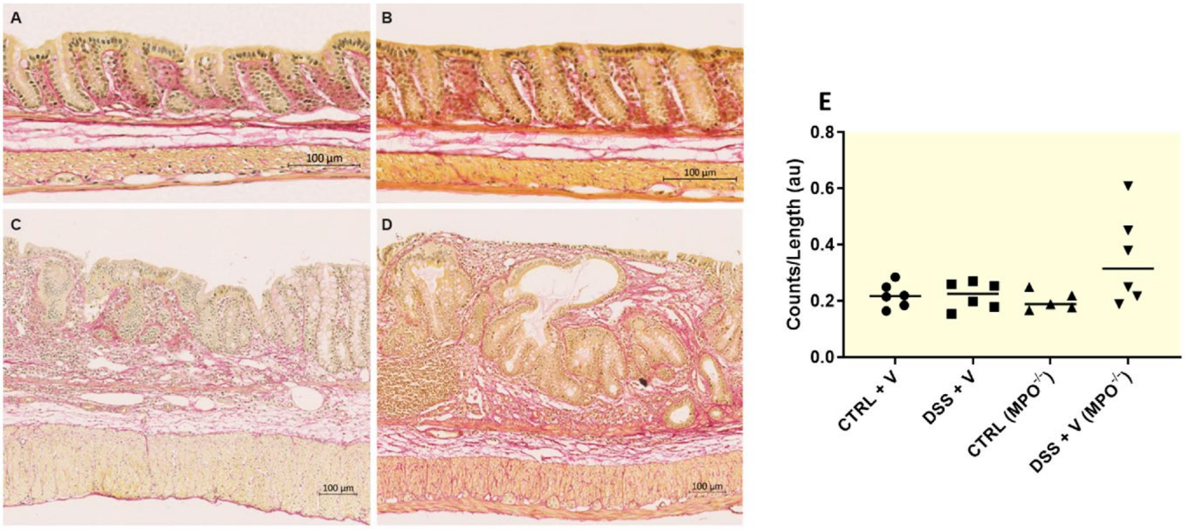

Supplementary Figure S3. Representative picrosirius red staining of colonic section stains with quantification. Representative colon sections stained with after 63-day chronic colitis model of groups A) Wt-CTRL, B) MPO KO-CTRL, Wt-DSS, C) and D) MPO KO-DSS. E) Quantitative assessment of positive picrosirius red staining of collagen in colons subjected to chronic DSS colitis. CTRL = control. DSS = dextran sodium sulfate. MPO KO = myeloperoxidase gene deficient. Data represented as mean ± SD. n = 6, all groups.

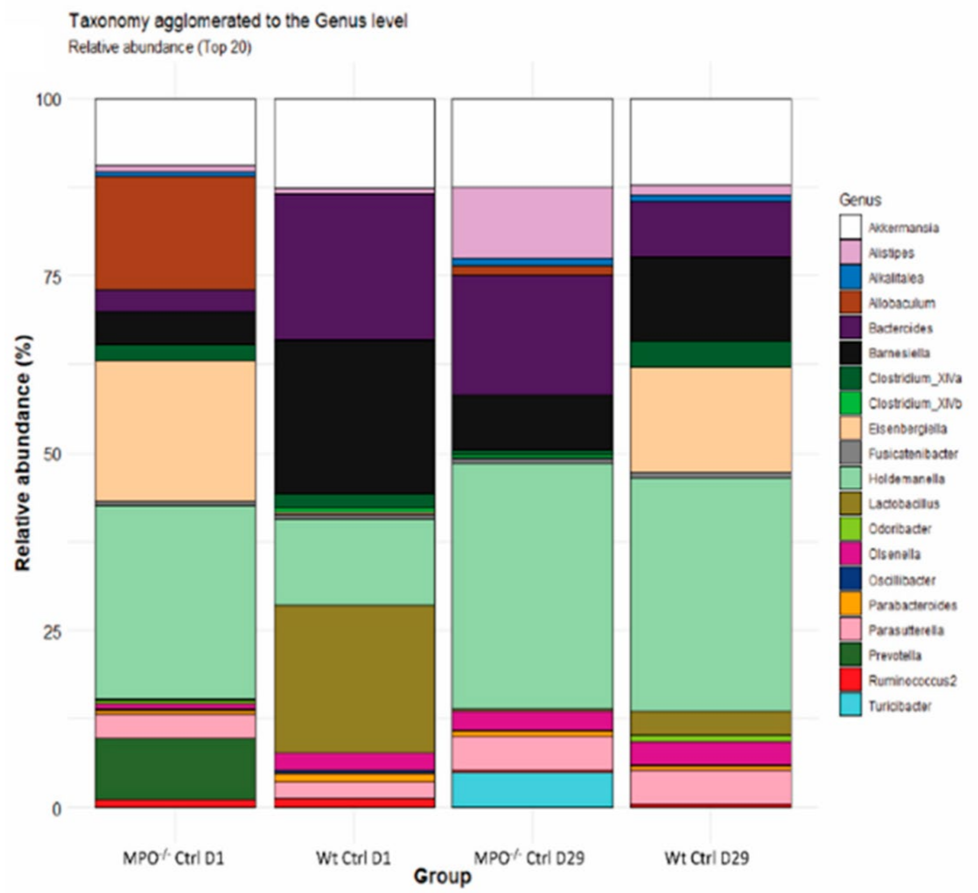

Supplementary Figure S4. Relative abundance of total bacterial genera in stool samples from Wt control and MPO KO control mice.

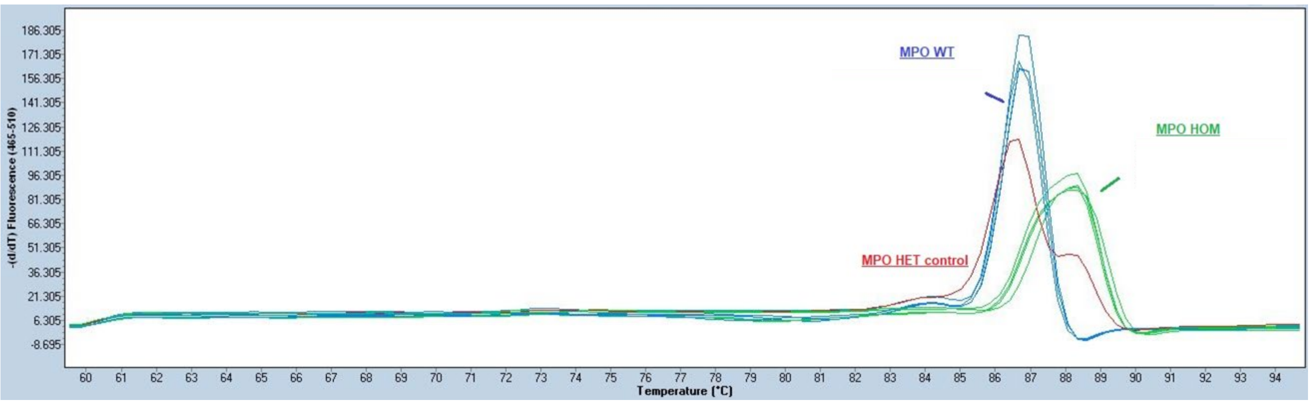

Supplementary Figure S5. RT-PCR high-resolution melting curve analysis of the MPO gene in randomly selected mice from Wt and MPO groups (n=3 per group). MPO WT = Wildtype mice, MPO HOM = Myeloperoxidase deficient mice, MPO HET control = Myeloperoxidase heterozygous control mice.

Table S1. Parameters assessed in colon histopathological scoring. Twenty random fields of view under 400x microscopic magnification were observed for colon sections stained with haematoxylin & eosin. A score between 0 and 2 was given for histopathological parameters including epithelium integrity, crypt integrity, level of goblet cell loss, extent of oedema and extent of leukocyte infiltration per field of view.

Score	Epithelium Integrity	Crypt Integrity	Goblet Cells	Extent of Oedema	Extent of Leukocyte Infiltration
0	Intact	Intact	Normal	Absent	None
0.5	Minimal damage	Mostly intact	Minor loss	Minimal	Mild
1	Partial shedding	Partial loss	Moderate loss	Submucosa only	Moderate; lamina propria
1.5	Severe damage	Mostly destroyed	Major loss	Lamina propria & submucosa	Substantial; lamina propria & submucosa
2	Destroyed	Entire crypt drop-out	Entire loss	Transmural	Extensive; transmural

Table S2. Antibodies and corresponding fluorochromes used in fluorescence-activated cell sorting. All antibodies obtained from Biolegend with the exception of GL3 from eBioscience.

**Target**	**Antibody Clone**	**Fluorochrome**	**Catalogue Number**
CD11b	M1/70	Allophycocyanin (APC)/Cyanine7 (Cy7)	101226
CD3	17A2	Alexa Fluor® 488 (AF488)	100212
CD4	GK 1.5	Peridinin-Chlorophyll-Protein (PerCP)/Cyanine5.5 (Cy5.5)	100434
CD45	30-F11	Brilliant Violet 785™ (BV785)	103149
F4/80	BM8	R-phycoerythrin (PE)/Cyanine7 (Cy7)	123114
gdTCR	GL3	PE/Cyanine5 (Cy5)	15-5711-82
I-Ab	AF6-120.1	Pacific Blue™ (PB)	116422
IL-17	TC11-18H10.1	PE	506904
Ly6C	HK1.4	Brilliant Violet 605™ (BV605)	128036
Ly6G	1A8	Alexa Fluor® 647 (AF647)	127610
